# Evaluation of mesenchymal stem cells in treatment of infertility in male rats

**DOI:** 10.1186/scrt521

**Published:** 2014-11-23

**Authors:** Amal I Hassan, Sally S Alam

**Affiliations:** Radioisotopes Department, Atomic Energy Authority, Giza, 12311 Egypt; Cell Biology Department, National Research Center, El Tahrir Street, 12622 Dokki Giza, Egypt

## Abstract

**Introduction:**

The present study aimed to elucidate the therapeutic effects of mesenchymal stem cells (MSCs) derived from the bone marrow of rats (BM) against toxic effects of lead (Pb) on the male gonads of experimental rats.

**Methods:**

The experimental animals were exposed to lead in the form of lead nitrate (LN) one quarter of the LD50. The efficacy of MSCs to reduce gonado-totoxicity induced by lead nitrate at 21, 30 and 60 days, was evaluated experimentally in male rats.

**Results:**

The results showed that testosterone levels and semen quality ameliorated following treatment with MSCs. Also, superoxide dismutase, glutathione peroxidase and catalase levels were increased 21, 30 and 60 days post treatment of MSCs. Moreover, a decrease in genomic DNA alteration and percentage of fragmented DNA was recorded after MSCs treatment. Lead nitrate caused degeneration, necrosis, interstitial edema, and reduction in spermatogenic activity in some seminiferous tubules. The LN-induced changes in histopathologic findings of testis were partially reversed by treatment with MSCs. Histological examination of testis showed deformities in morphology of testis in test animals with gross damage within the seminiferous tubules in Lead nitrate group. The LN-induced changes in histopathologic findings of testis were partially reversed by treatment of MSCs.

**Conclusions:**

It was concluded that lead is a gonadotoxic with a tendency of suppressing semen characteristics and testosterone levels of animals, the presence of MSCs was found to alleviate the toxic effects of lead. We conclude that MSCs derived from the bone marrow of rats can be an effective therapy of LN induced gonado toxicity, thus can contribute to the treatment of infertility.

## Introduction

Metals are unique environmental toxicants as they tend to possess bioaccumulative, immutable and non-biodegradable properties and pose a serious threat to eco-biological systems [1]. Lead (Pb) is one of the well-known ubiquitous non-essential metals with wide applications for many centuries, which is released into the environment by several routes, but principally by industrial, mining and hunting activities [2]. Exposure to lead is implicated in serious health hazards in animals and humans due to its toxicity and its ability to accumulate in living organisms [3]. The deterioration of male reproductive health is one of the major manifestations of occupational and/or environmental exposure to Pb toxicity [1]. Earlier studies have demonstrated that lead can pass through the blood-testis barrier, accumulate in the testis and/or epididymis and affect the germinal cells at different levels of differentiation (spermatogonia, primary spermatocytes, spermatids or spermatozoa) [4]. Lead-exposed battery factory workers have shown a decrease in sperm count, density, motility and semen volume [5, 6]. In addition, studies of Biswas and Ghosh [7] demonstrated that lead exposure reduces the activity levels of testicular steroidogenic enzymes in rats.

Some studies suggested that oxidative stress is a potential contributor to lead toxicity and that lead directly or indirectly changes the pro-oxidant and antioxidant balance in the biological system by the generation of more reactive oxygen species (ROS), which elicits oxidative damage of proteins, lipids and DNA [8–10]. Antioxidant defenses, such as catalase (CAT), superoxide dismutase (SOD) and glutathione reductase (GR), are involved in counteracting the toxicity of ROS [11]. Under normal conditions, these antioxidants protect the cells and tissues from oxidative damage. Enhanced generation of ROS can overwhelm cells intrinsic antioxidant defenses and result in a condition known as ‘oxidative stress’. Cells under oxidative stress display various dysfunctions due to lesions caused by ROS to lipids, proteins and DNA. Consequently, it has been suggested that metal-induced oxidative stress in cells can be partially responsible for the toxic effects of heavy metals [12].

Bone marrow stem cells, including hematopoietic stem cells and bone marrow-derived mesenchymal stem cells (MSCs/BM), are pluripotent and can self-renew. MSCs/BM are characterized by their accessibility, ease of culture and proliferation *in vitro*, potential to modulate tissue repair and biological stability in long-term culture [13]. In addition, MSCs have been adapted in andrology research on erectile dysfunction and infertility as potential therapeutic agents. The studies related to this area showed that MSCs derived from human fetal lung and umbilical cord can differentiate into sperm-like cells [14, 15]. The differentiation of MSCs into germ cells, Sertoli cells and Leydig cells was also demonstrated in busulfan-treated infertile mice [16]. These insights hold promise to inform strategies for the directed differentiation of stem cells and to offer the potential for novel metabolic or pharmacological therapies to enhance regeneration and the treatment of degenerative disease [17].

Recently, the focus in stem cell biology has been on the adverse effects of ROS, particularly the damaging effects of ROS accumulation on tissue aging and the development of cancer. Various anti-oxidative and anti-stress mechanisms of stem cells have also been characterized [18, 19].

The effects of MSCs on lead-induced reproductive toxicity have not yet been reported. The present study was undertaken to investigate the ability of MSCs to modify (1) the oxidative stress and (2) the suppressed reproduction induced by lead in male rats.

## Materials and methods

### Ethics statement

Anesthetic procedures and handling of animals were approved by, and complied with, the ethical guidelines of the Medical Ethical Committee of the National Research Centre in Egypt (Approval number: 10031).

### Animals

Adult male albino rats weighing 160 ± 10 g were used in the present study. The animals were kept in wire bottomed cages in a room under standard conditions of illumination with a 12-hour light–dark cycle at 23 ± 1°C. They were provided with tap water and a balanced diet *ad libitum*.

### Preparation of bone marrow-derived MSC (MSCs/BM)

**Figure 1:**
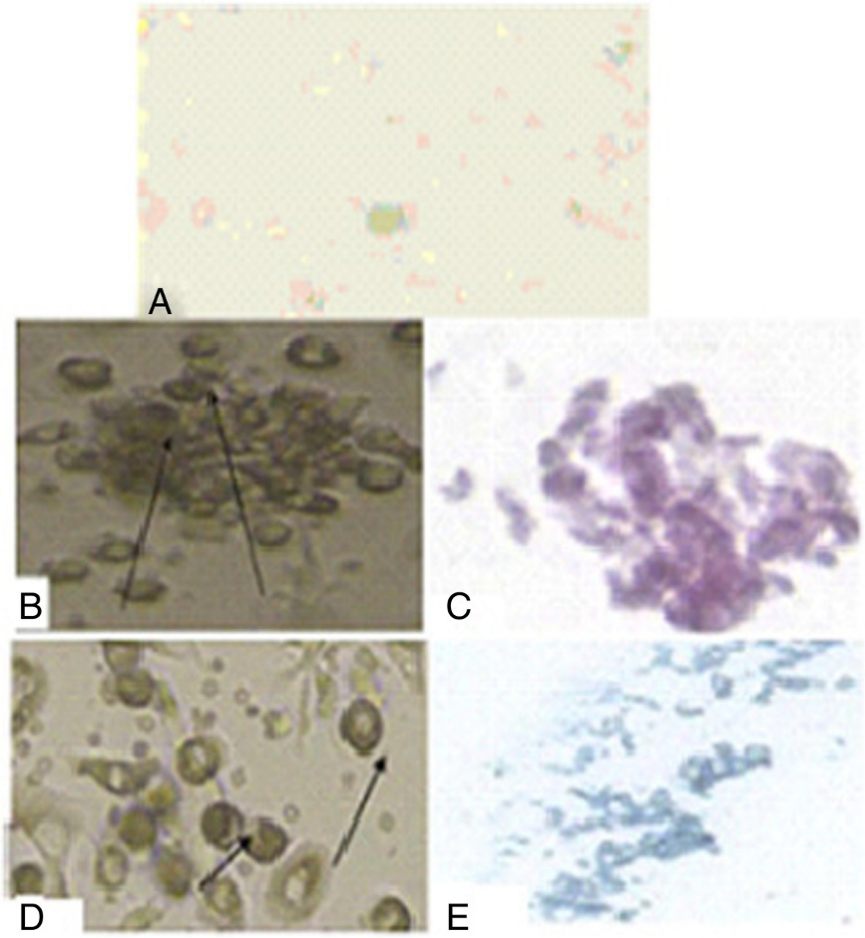
**Morphological and histological staining of differentiated MSCs/BM. A**. Undifferentiated MSCs. **B**. Differentiated MSC osteoblasts after addition of growth factors. **C**. MSCs differentiated into osteoblasts stained with Alizarin red. **D**. Arrows for differentiated MSC chondrocytes after addition of growth factors. **E**. MSCs differentiated into chondrocytes stained with Alcian blue. MSCs/BM, bone marrow-derived mesenchymal stem cells.

**Figure 2:**
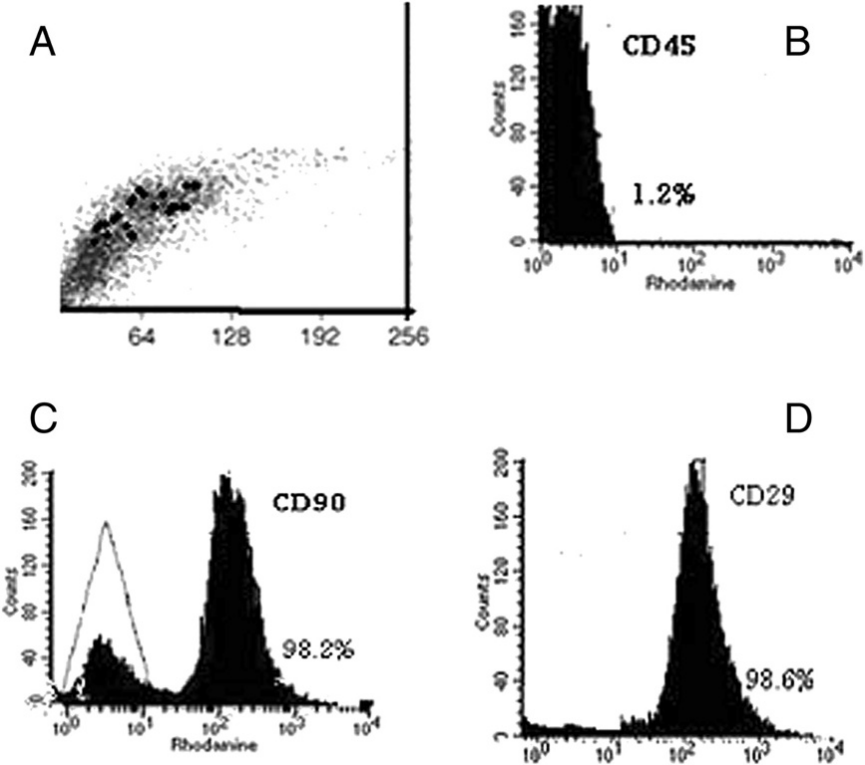
**Characteristics of MSCs/BM.** Cells were stained with the CD45, CD90 and CD29 antibodies and analyzed by flow cytometry. **A)** BM-MSCs are shown as a dot plot. **B)** The expression levels of CD45-ve, **C)** CD90 + ve and **D)** CD29 + ve of BM-MSCs are presented as a histogram. MSCs/BM, bone marrow-derived mesenchymal stem cells.

Bone marrow was harvested by flushing the tibiae and femurs of male albino rats with (Dulbecco’s) modified Eagle’s medium ((D)MEM, Gibco BRL, Life Technologies, Grand Island, NY, USA) supplemented with 10% fetal bovine serum (GIBCO/BRL). Nucleated cells were isolated with a density gradient (Ficoll/Paque (Pharmacia, Uppsala, Sweden)) and resuspended in complete culture medium supplemented, then incubated at 37°C in 5% humidified CO_2_ for 12 to 14 days as the primary culture or until formation of large colonies. When large colonies developed (80% to 90% confluence), the cultures were washed twice with phosphate-buffered saline (PBS) and the cells were trypsinized with 0.25% trypsin in 1 mM ethylenediaminetetraactic acid (EDTA) (GIBCO/BRL) for five minutes at 37°C. After centrifugation, the cells were resuspended with serum-supplemented medium and incubated in 50 cm^2^ culture flasks (Falcon, Pharmacia, Uppsala, Sweden). The resulting cultures were referred to as first-passage cultures [20]. MSCs in culture were characterized by their adhesiveness and fusiform shape [21]. The resulting cultures were referred to as first-passage cultures [22]. On day 14, the adherent colonies of cells were trypsinized and counted. Cells were identified as being MSCs by their morphology, adherence, and power to differentiate into osteocytes [23] (Figure 1B,C) and chondrocytes [24] (Figure 1D,E). Differentiation into osteocytes was achieved by adding 1 to 1,000 nM dexamethasone, 0.25 mM ascorbic acid, and 1 to 10 mM beta-glycerophosphate to the medium. Differentiation of MSCs into osteoblasts was confirmed through morphological changes, Alzarin red staining of differentiated osteoblasts and RT-PCR gene expression of osteonectin in differentiated cells. Differentiation into chondrocytes was achieved by adding 500 ng/mL bone morphogenetic protein-2 (BMP-2; R&D Systems, Minneapolis, MN, USA) and 10 ng/ml transforming growth factor β3 (TGFβ3) (Peprotech, London, UK) for three weeks [24]. After passage 3 (P3), stem cells were harvested. Immunophenotyping using 100 ml of the cell suspension was performed by flow cytometry (Accuri, BD Accuri C6; Becton Dickinson San Jose, CA, USA). The MSCs are positive for CD29 (Sigma, San Diego, CA, USA, SAB 4501582) and negative for CD45 (Sigma, OX-1 84112004) (Figure 2A-D).

### Experimental design

Seventy male rats were randomly divided into two main groups as follows: (1) the normal control group (GI) (N = 10): (normal saline (NS) received 0.4 ml of physiological saline via the right tail vein; and (2) the lead treated group (LN) (GII) (N = 60): animals were injected intravenously (through the tail vein) with a single dose of lead nitrate (LN, 23.3 mg/kg body weight (bw)) about ¼ LD50 according to the Agency for Toxic Substance and Disease Registry (ATSDR) [25]. GII was divided into two subgroups, GII(A) treated with 1 × 10^6^ MSCs per rat [22] one week after a single dose of LN (23.3 mg/kg bw) about ¼ LD50 (N = 30) while GII(B) animals did not receive MSCs (N = 30). Ten animals only were tested at 21, 30 and 60 days, respectively, post-injection.

### Preparation of tissue homogenate

The excised testicular tissue was washed with distilled water for the removal of blood, after which the fatty parts were removed. Tissues were homogenized in ice-cold 50 mM sodium phosphate buffer (pH 7.4) containing 0.1 mM EDTA. The supernatant was separated by centrifugation at 1,000 g for 20 minutes at 4°C. The supernatants were used for the analysis of all antioxidant enzymes.

### Estimation of lipid peroxidation and assay of antioxidant enzymes in testes

Malondialdehyde (MDA) in the testis homogenate was assayed colorimetrically according to the method of Ohkawa *et al*. [26], in which MDA is determined by using 1 ml of trichloroacetic acid (10%) and 1 ml of thiobarbituric acid (0.67%).

SOD was determined according to the method described by Misra and Fridovich [27]. The activity of CAT, expressed as units/mg protein, was measured spectrophotometrically at 240 nm by calculating the rate of degradation of H_2_O_2_, the substrate of the enzyme [28]. The determination of testicular glutathione peroxidase (GPx) activity was carried out according to the method of Chiu *et al*. [29].

### Analysis of sperm parameters

Epididymal sperm were obtained by chopping cauda epididymis in 5.0 ml of Ham’s F12 medium. The sperm were counted using a Neubauer Chamber as describe by Belsey *et al*. [30]. Progressive sperm motility was evaluated by a previously described method [30] within five minutes following their isolation from cauda epididymis at 37°C and the data were expressed as percent motility. The morphological abnormalities in sperm were enumerated by the methodology reported by Hemavathi and Rahiman [31] using light microscopy.

### Genetic analysis

#### DNA extraction

Isolation of genomic DNA from caudal epididymal spermatozoa was carried out using the protocol of Gebert *et al*. [32]. Briefly, sperm cells were lysed in 500 μl of a buffer consisting of 50 mM Tris–HCl at pH 8.0, 100 mM NaCl2, 100 mM EDTA, 1% SDS and treated with 2.5 μl of Triton X-100 (Merck, Darmstadt, Germany), 21 μl of dithiothreitol (1 M) (Sigma-Aldrich Chemie) and 40 μl of proteinase K (10 mg/ml). DNA precipitation was performed in a saturated sodium chloride solution with subsequent addition of 100% ethanol (Roth, Hamburg, Germany).

The concentration of DNA and its relative purity were determined using a spectrophotometer based on absorbance at 260 and 280 nm, respectively. The integrity of extracted genomic DNA was verified by electrophoresis in 0.8% agarose gel using a DNA molecular weight marker (Eurblio, Paris, France).

#### Random amplified polymorphic DNA analysis

DNA from samples was used for random amplified polymorphic DNA (RAPD) analysis following the method recommended by [33]. The cocktail for the amplification was prepared as follows in 25 ul PCR tubes: genomic DNA 50 ng/ml, 25 pmol dNTPs, and 25 pmol of random primer, 0.8 units of *Taq* DNA polymerase. A set of four 10-mer primers (Operon Technologies Inc., Alameda, CA, USA) randomly selected were used in the RAPD analysis (Table 1). The reaction mixture was given a short spin to thoroughly mix the cocktail components. Then, the PCR tubes were loaded onto a thermal cycler (Perkin-Elmer 9700) programmed with a first denaturation of five minutes at 94°C, followed by 45 cycles of one minute denaturation at 95°C, one minute annealing at 36°C and two minutes extension at 72°C. A final extension at 72°C for five minutes was allowed before holding the reaction at 4°C for ten minutes. Reaction products were stored at 4°C prior to electrophoresis. The products (15 μl each), mixed with 3 μl loading buffer (0.25% bromophenol blue, 0.25% xylene cyanol and 30% glycerol in water), were loaded on 2% agarose gels and electrophoresed at 100 V for one hour. A DNA marker (Thermo Scientific, Operon Technologies, Alameda, CA, USA) was used as a size comparison. The DNA marker contained a total of ten fragments ranging from 100 bp to 1,000 bp in 100 bp increments. Amplification products separated by gels were visualized and documented using the Gel Documentation system, XR^+^ Molecular Imager apparatus (BIO-RAD, Poland). Polymorphism was defined as the presence and/or absence of DNA fragments between the samples.

**Table 1:** **Sequence of selected random primers, number of total bands and percentage of polymorphisms calculated from treated and control sperm cells**

Primers	Sequence (5′- 3′)	GC%	Total number of band studied	Number of polymorphic bands	Polymorphism (%)	Size range (bp)	
						Max.	Min.
OPA01	CAG GCC CTTC	70	11	9	81.8	1097	63
OPA08	GTG ACG TAGG	60	8	4	50	692	200
OPA12	TCG GCG ATAG	60	13	11	84.6	1301	130
OPA20	CTT GCG ATC C	60	12	11	91.6	874	84
Total			44	35	79.5	1301	63

#### Agarose gel electrophoresis for DNA fragmentation

DNA was isolated from rat testis using proteinase K and RNase A with the methods of Gilbert *et al*. [34]. To estimate DNA damage, 5 μg of rat DNA was separately loaded on 1.5% agarose gel containing 1.0 μg/ml ethidium bromide including DNA standards (0.5 μg per well). Electrophoresis was performed for 45 minutes at 100 volts. After electrophoresis, the gel was studied under a gel doc system and was photographed with a digital camera.

### Testosterone measurement

At 21, 30 and 60 days post treatment, blood samples were collected from the retroorbital plexus in plastic centrifuge tubes, left to clot at 4°C for 30 minutes and serum obtained by centrifugation at 3,000 rpm for 20 minutes. Quantitative measurement of serum testosterone was carried out by solid phase radioimmunoassay (RIA) using components of a commercial kit (Coat-A-Count, Siemens Medical Solutions Diagnostics, Los Angeles, CA, USA) with modifications described by Richards *et al*. [35].

### Serum protein assay

The total protein level was determined according to the method of Bradford [36].

### Histological examination

Pieces of testes were fixed in 10% neutral-buffered formalin that was embedded in paraffin and the deparaffinized sections were stained routinely with hematoxylin and eosin as described by Bancrofet and Stevens [37].

### Statistical analysis

All values are expressed as mean ± SE. Statistical analysis was performed with two way analysis of variance (ANOVA) followed by Duncan’s test. *P* values <0.05 were considered to be statistically significant.

## Results

### Testicular oxidative stress

**Figure 3:**
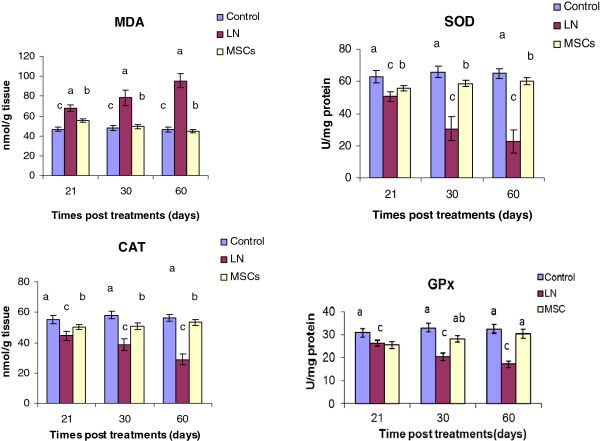
**Effect of treatments on testicular lipid peroxidation and antioxidant enzymes after 21, 30 and 60 days of exposure.** MDA: malondialdehyde; SOD: superoxide dismutase; CAT: Catalase; GPx: glutathione peroxidase.

The effects of LN exposure on testicular MDA and antioxidant related parameters and their response to MSCs after 21, 30 and 60 days of exposure were assessed and are presented in Figure 3. The results revealed that LN produced a statistically significant increase in the level of testicular content of MDA and a significant decrease in SOD, CAT and GPx activity at different time intervals of exposure, in comparison to the control group. However, treatment with MSCs along with LN caused a significant reduction in the MDA level when compared with the lead only group. A significant increase in the activity of SOD, CAT and GPx was observed after the treatment with MSCs, in comparison to LN exposed group (Figure 3).

LN exposure significantly decreased (*P <* 0.05) total protein level at 21, 30 and 60 days. Meanwhile, after 30 and 60 days of MSCs treatment the level of total protein was increased significantly as compared to the untreated rats (Figure 3).

### Testosterone level and total protein

**Figure 4:**
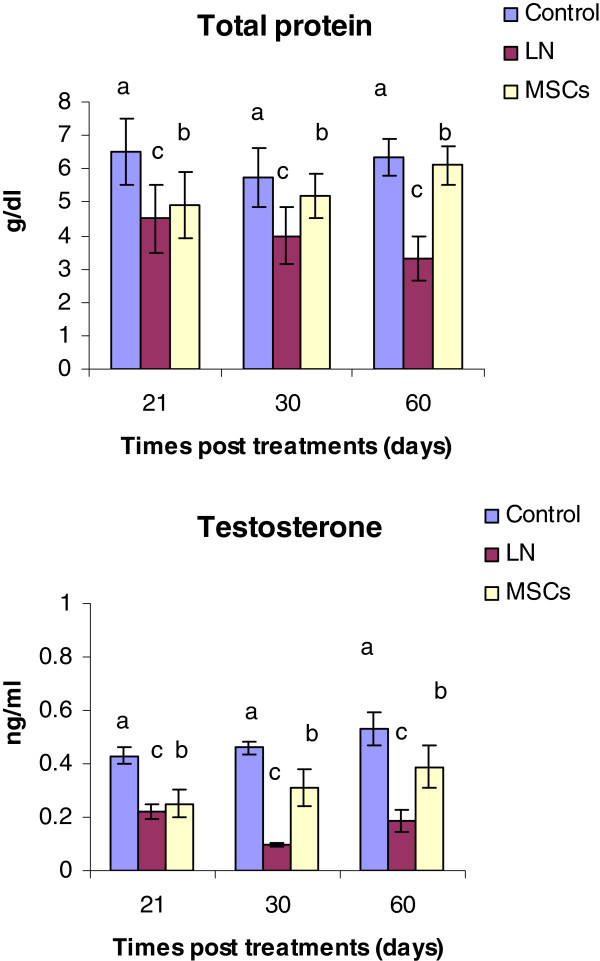
**Effect of treatments on serum testosterone and total protein levels after 21, 30 and 60 days of exposure.**

As shown in Figure 4, LN caused a significant decline (*P <* 0.05) in testosterone level (0.22 ± 0.05, 0.095 ± 0.007 and 0.17 ± 0.004) at 21, 30 and 60 days, respectively, after treatment as compared to control rats (0.43 ± 0.02). The data revealed that MSCs modulated this decline of serum testosterone level post 21, 30 and 60 days and approached control values, especially after 60 days (0.388 ± 0.06). LN exposure significantly decreased (P<0.05) total protein level at 21, 30 and 60 days. Meanwhile, after 30 and 60 days of MSCs treatment the level of total protein was increased significantly as compared to the untreated rats (Figure 3).

### Sperm analysis

The effects of lead and co-administration of MSCs on total epididymal sperm count, sperm motility and sperm abnormalities are shown in Table 2. The mean percentage of sperm count and sperm motility for animals treated with LN significantly decreased compared to control animals. A significant increase in sperm shape abnormalities was detected after LN exposure. These effects were time dependent. Treatment with MSCs significantly improved the sperm count and motility.

**Table 2:** **Lead nitrate-induced changes in sperm characteristics and their response to administration of MSCs/BM in rats after 21, 30 and 60 days of exposure**

Groups	Parameters	Mean control value	LN			LN + MSCs/BM		
			21 days	30 days	60 days	21 days	30 days	60 days
**Sperm count (10** ^**6**^ **/ml)**		110.5^a^ ±2.48	52.44^d^ ±1.83	66.5^cd^ ±2.14	82.3^bc^ ±2.35	89.85^b^ ±3.11	97.65^b^ ±2.58	107.93^ab^ ±4.01
**Sperm motility (%)**		98.73^a^ ±2.33	44.06^d^ ±1.25	50.41^c^ ±1.62	72.5^b^ ±0.48	81.61^a^ ±2.55	90.48^a^ ±1.54	96.86^a^ ±2.43
**Abnormal sperm (%)**		9.75^f^ ±1.42	63.78^a^ ±3.0	48.55^b^ ±2.31	35.23^c^ ±2.41	20.72^d^ ±1.33	11.25^e^ ±1.80	9.09^f^ ±0.75
**Sperm morphology (%)**		91.25^a^ ±1.35	36.22^d^ ±0.05	51.45^c^ ±0.07	64.77^c^ ± 0.11	79.28^b^ ±2.33	88.75^ab^ ±2.90	90.91^a^ ±2.00

Values are mean ± S.E. of 10 individuals. Mean values with same superscripts do not differ significantly from each other. *P* <0.05. LN, lead nitrate; MSCs/BM, bone marrow-derived mesenchymal stem cells; S.E., standard error.

### Random amplified polymorphic DNA-PCR

To analyze instability in the genome of treated rats compared to non-treated controls using RAPD-PCR fingerprinting, four random 10-mer primers were used to amplify genomic DNA samples. RAPD primers generated strong banding patterns in all samples tested, and the presence of changes in the RAPD profiles obtained from the exposed animals depended on the primer used. Profiles generated by these primers revealed differences between control and exposed rats, with visible changes in the number and size of amplified DNA fragments.

The RAPD profiles obtained with the RAPD primers exhibited bands between 63 to 1,301 bp in length. In a total of 44 bands scored, 35 bands were polymorphic giving 79.5% polymorphism. Primers OPA08 and OPA12 amplified the minimum and maximum number of bands which were 8 and 13 bands, respectively.

**Figure 5:**
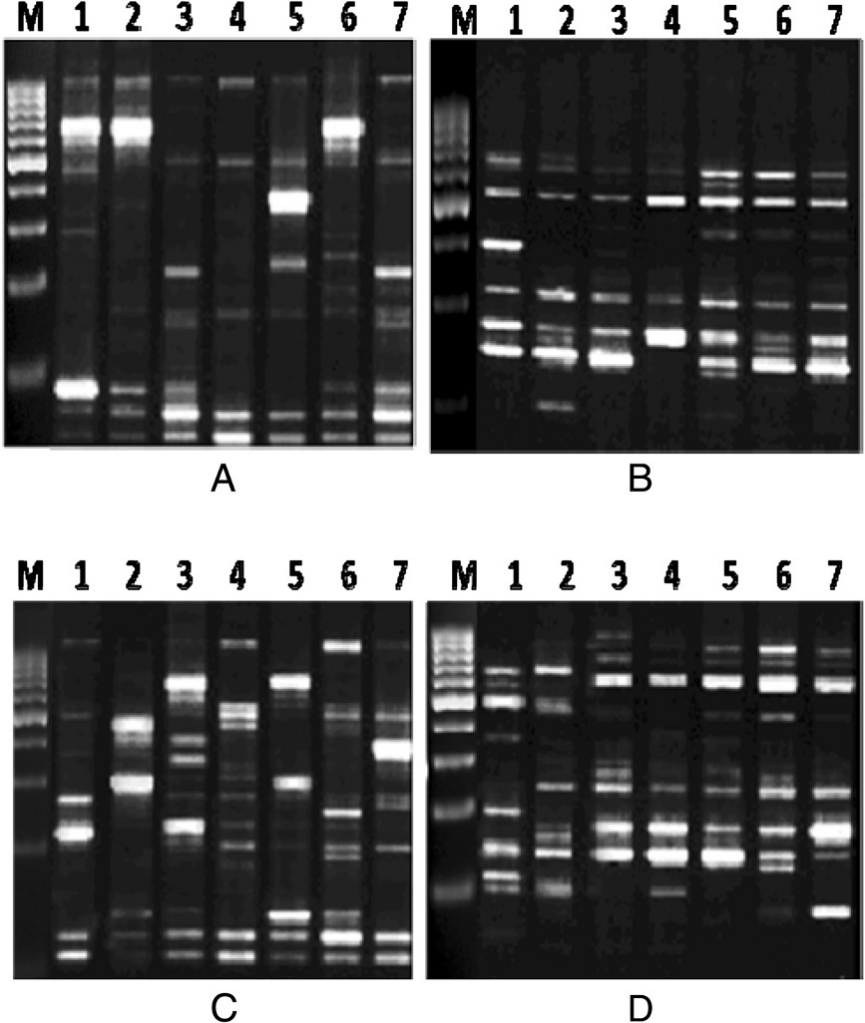
**RAPD-PCR fingerprinting of sperm generated by primers OPA01 (A), OPA08 (B), OPA12 (C) and OPA20 (D).** The appearance and disappearance of bands of the same pattern as the negative control were considered. Rat samples treated with lead nitrate after 21 days (Lane 7), 30 days (Lane 5) and 60 days (Lane 3). Rat samples treated with both lead nitrate and MSCs/BM after 21 days (Lane 6), 30 days (Lane 4) and 60 days (Lane 2). Normal control group (Lane 1). M = marker 1,000 bp. MSCs/BM, bone marrow-derived mesenchymal stem cells; RAPD, random amplified polymorphic DNA.

**Figure 6:**
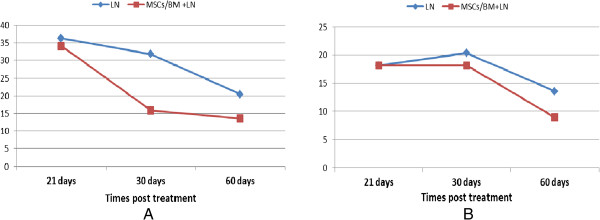
**Genomic damage.** The percentage of altered bands in each treatment detected by RAPD-PCR. **A**. Average band gains. **B**. Average band loss. RAPD, random amplified polymorphic DNA. LN: Lead nitrate; MSCs/BM: Mesenchymal stem cells derived from bone marrow; DNA: Deoxyribonucleic acid.

The total number of amplification products generated by these individual primers and variable fragments are described in Table 1, and representative RAPD fingerprints are shown in Figures 5A-D.Alterations in RAPD ‘fingerprints’ produced by the random primer set were seen in one of two ways. There was the gain or loss of a band. The quantitative analysis of those bands, expressed as percentage of band loss and gain, shows a time-dependent relationship. In the case of band gain, at 21 days after lead treatment 16 new bands were amplified, representing 36.3%, while at 30 and 60 days after lead treatment, 14 and 9 new bands were amplified, representing 31.8% and 20.4%, respectively. The trend of increase in band gain as related to the decrease in time after lead exposure is depicted in Figure 6A.Similarly, in the case of band loss, at 21 days after lead treatment eight bands had disappeared representing 18.2% (Figure 6B). At 30 and 60 days, nine and six bands had disappeared representing 20.4% and 13.6%, respectively.Treatment with stem cells ameliorated the effects of lead at different time intervals as evidenced by a decrease in the percentage of polymorphic bands (loss and gains) as shown in (Figures 6A and B).

### Effect of stem cells and lead on DNA fragmentation

**Figure 7:**
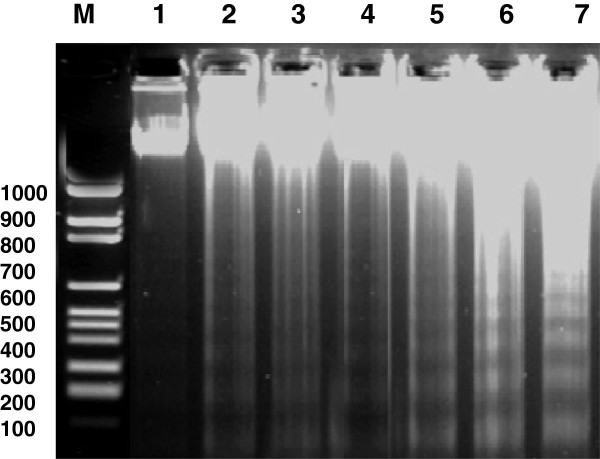
**Agarose gel electrophoresis of DNA extracted from rat testis in different groups: after 21 days (lane 7), 30 days (lane 5) and 60 days (lane 3) of lead nitrate exposure and treatment with both lead nitrate and MSCs/BM after 21 days (lane 6), 30 days (lane 4) and 60 days (lane2); normal control group (lane1).** M: marker 100- to 1,000 bp. MSCs/BM, bone marrow-derived mesenchymal stem cells.

DNA fragmentation in response to lead exposure was detected by gel electrophoresis as a DNA ladder representing a series of fragments that are multiples of 180 to 200 bp (Figure 7). The diffuse pattern of DNA degradation was increased after 21 days than 30 days and 60 days of lead treatment. In contrast, treatment with stem cells led to significant protection against lead-induced DNA fragmentation. On the other hand, the control group did not reveal any damage to DNA.

### Histopathological results

**Figure 8:**
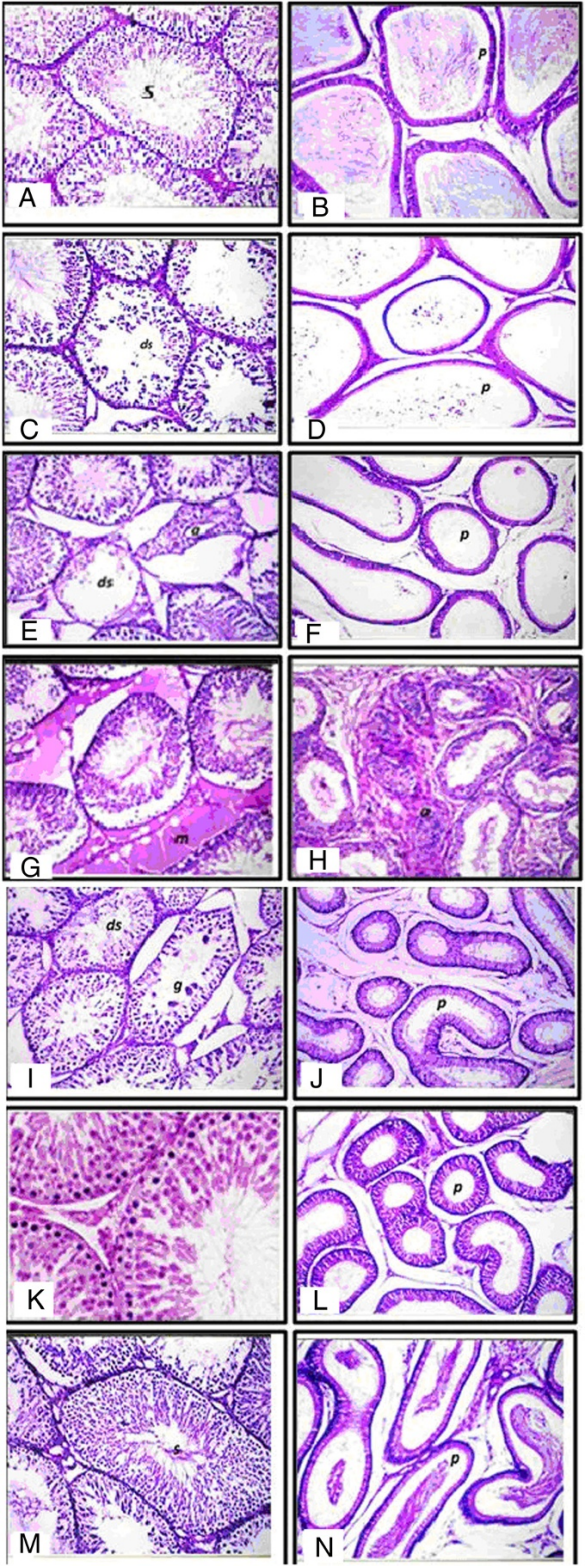
**Histopathology showing the alterations induced by LN and treatment effects of MSCs/BM in testis tissues of rat.** Photomicrographs of H & E-stained sections of **(A)**: testes of control rats showing normal histological structure of the mature active seminiferous tubules with complete spermatogenic sense (s) × 40. **B**: epididymis of rats in the control group showing normal histological structure of the tubules and impacted by mature sperm (*p*) × 40. **C**: photomicrographs of H & E stained sections of LN groups after 21 days showing degeneration in some seminiferous tubules (ds) × 40. **D**: epididymus of rats in the LN group post 21 days showing epididymal tubules lumen (*p*). **E** and **F**: section of testes of rat in the LN group post 30 days showing degeneration (ds) and atrophy (a) of some individual seminiferous tubules (8E), and epididymal tubules free from sperm (*p*) (8 F). **G** and **H**: section of the testes rat post 60 days of LN showing homogenous eosinophilic structure material replacing the interstitial of Leydig cells (m) (8G) and anaplastic activity in the lining epithelium of the epididymal tubules (a) with empty lumen (8H). **I and J**: photomicrographs of testes post 21 days of MSCs/BM showing degeneration (ds) with giant spermatogonial formation (g) of some seminiferous tubules (8I) and epididymal tubular lumen (p) with swelling in the lining epithelium × 40 (8 J). **K** and **L**: section of testes post 30 days of MSCs/BM treatment showing mild degeneration in some seminiferous tubules (ds) (8 K) with spermatogonial formation (8 L). **M** and **N**: testes of rat post 60 days of MSCs/BM treatment showing normal intact histological structure with complete active seminiferous tubules sense (s) (8 M) and intact normal histological structure of the epididymal tubules impacted by spermatozoa in the lumen (*p*) × 40 (8 N). LN, lead nitrate; MSCs/BM, bone marrow-derived mesenchymal stem cells.

There was no histopathological alteration observed and the normal histological structure of the tubules with multiple sperm in the tubular lumen is shown in Figure 8A and B. Degenerative change was detected in some seminiferous tubules and the tubular lumens were empty of spermatozoa 21 days after LN (Figure 8C and D). Degeneration and atrophy were also detected in some seminiferous tubules and the tubular lumens were empty of spermatozoa in most examined seminiferous tubules 30 days after LN exposure (Figure 8E and F). Homogenous eosinophilic material broadly replaced the interstitial of Leydig cells 60 days after LN exposure (Figure 8G). Moreover, the lining epithelium showed anaplastic activity while the tubular lumen was empty of sperm 60 days after LN (Figure 8H). In examination of sections of testes post 21 days treatment with MSCs, degeneration with giant spermatogonial cells was observed in some seminiferous tubules (Figure 8I). The tubular lumens were empty of spermatozoa associated with swelling in the lining epithelium (Figure 8J). In animals treated with MSCs, mild degeneration was detected after 30 days in a few seminiferous tubules (Figure 8K and L). There was no histopathological alteration recorded post 60 days of MSCs treatment (Figure 8M and N).

## Discussion

The effect of environmental lead on the male reproductive system in which the testicular spermatogenesis and spermatozoa within the epididymis are the major targets for lead action to produce toxicity on reproduction has been a major area of concern for several years [38]. Although the exact mechanism of LN-induced toxicity is not completely understood, cumulative data has shown that oxidative stress plays an essential role in its toxicity. Lead administration disrupts the testes spermatogenesis process via mechanisms that involve the induction of lipid peroxidation, depletion of ROS scavengers and disruption of testicular antioxidant enzyme activity [39].

In the present study, lead exposure correlated with increased levels of oxidative stress biomarkers in the testis of rats, represented by decreased levels of antioxidant enzymes (SOD, CAT, and GPx) and an increase of testicular MDA. MDA is one of the major products of peroxidized polyunsaturated fatty acids and increased MDA content is an important indicator of lipid peroxidation. In general, SOD is the first line of defense against oxidative stress [40] and plays a pivotal role in dismutation of superoxide anions to hydrogen peroxide, and CAT neutralizes hydrogen peroxides to molecular oxygen and water [41]. The decrease in these enzymes in Pb treated rats clearly postulates improper dismutation of superoxides and improper decomposition of H_2_O_2_. The production of ROS is a normal physiological event in various organs including the testis. On the other hand, overproduction of ROS can be harmful to sperm and, subsequently, to male fertility [42]. It has been demonstrated that lead toxicity leads to free radical damage via two separate pathways [43]: 1) the generation of ROS, including hydroperoxides, singlet oxygen and hydrogen peroxide; and 2) the direct depletion of antioxidant reserves [12]. Marchlewicz *et al*. [39] and Sainath *et al*. [44] demonstrated that Pb toxicity caused testicular oxidative stress by increasing the levels of lipid peroxidation and decreasing the activities of SOD and CAT in testes, which is inconsistent with the findings of the present study.

However, administration of MSC/BM extract significantly prevented the influence of lead on the antioxidative system. It decreased MDA and concomitantly increased the activities of SOD, CAT and GPx levels in testes tissue. These results clearly demonstrate the anti peroxidative role of the MSC/BM. The *in vivo* protection by MSC/BM against lead-induced oxidative damage may be because of its free radical scavenging potential. The specific responses of MSCs to oxidative stress may play a crucial role in the regulation of tissue homeostasis as well as regeneration of organs after oxidative injury [45]. It could also be because of direct scavenging/neutralization of the free radical or induction of endogenous antioxidant enzymes, such as CAT and SOD. El Far *et al*. [46] reported that transplantation of MSCs can correct and reverse the imbalance between ROS and antioxidant defense in favor of antioxidant defense by restoring and augmenting its capacity as well as modulating lipid peroxidation.

The sperm endpoints, such as epididymal sperm count and sperm motility, were used as important indicators to detect adverse effects of various factors on spermatogenesis [47]. In the present study, epididymal sperm count and sperm motility decreased in rats at different time intervals of exposure to lead as compared to control rats. These results are in agreement with earlier reports [48]. Moreover, Leiva *et al*. [49] reported a reduction in epididymal sperm number and daily sperm production in male rats treated with lead acetate and explained this reduction in sperm number by positing that lead acetate administration inhibited spermatogenesis by reducing the length of the stages related to spermiation and onset of mitosis. The deterioration in the selected sperm characteristics might be due to increased oxidative stress during Pb intoxication. Oliveira *et al*. [50] showed that lead had adverse effects on the sperm in male rabbits. It increased the percentage of sperm shape abnormalities. These findings coordinate with results reported by those who found a decrease in the percentage of sperm motility and intact acrosomes in mice treated with lead acetate. Also, Mendiola *et al*. [51] reported a significant positive association between the percentage of immotile sperm and seminal plasma levels of lead and cadmium in men. Moreover, Leiva *et al*. [49] reported a reduction in epididymal sperm number and daily sperm production in male rats treated with lead and explained this reduction in sperm number by the fact that lead acetate administration inhibited spermatogenesis by reducing the length of the stages related to exposure to lead and induction of genetic damage. Spermiation and onset of mitosis [49, 52, 53] indicated that lead acts as a spermicidal agent in the case of high exposure for a long time. Our experiments showed that the toxic effects of lead on the reproductive system in male rats were dose-dependent.

Poor sperm quality caused by oxidative stress due to generation of ROS has been reported to result in infertility [54]. Several studies suggest a correlation between increased ROS production and decreased sperm motility [55, 56]. It is hypothesized that H_2_O_2_, one of the lipid peroxidation products, might diffuse across the membrane and affect the vital enzymes in the sperm [57], thereby resulting in decreased sperm motility. Spermatogenesis occurs in the testis and its duration varies among species, for example, about 52 days in rats [58] and 64 days in humans [59]. Spermatozoa are generated in the testes and are transported to the epididymis for concentration and maturation. Spermatogenesis is a prolonged process spanning 40 to 50 days in rodents [59]. Agarwal *et al*. [60] and Manivannan *et al*. [61] showed that from 20 to 60 days the seminiferous tubules are in an active developing stage with cellular units in which the sections exhibited round-shaped seminiferous tubules and many newly formed spermatogenic cells arranged properly inside the tubules At 60 days, all stages of spermatogenesis were clearly visible and spread in the lumen of the tubules as was seen in the corresponding control. The total duration of spermatogenesis, which takes approximately 4.5 cycles, lasts from 30 to 75 days in mammals [58, 60]. Although strain or breed differences can be found in the literature among members of the same species [62], the duration of the spermatogenic cycle has been generally considered constant for a given species. According to a study utilizing xenogenic spermatogonial transplantation has demonstrated that the spermatogenic cycle duration is under the control of the germ cell genotype [58].

Spermatozoa are generated in the testes and are transported to the epididymis for concentration and maturation. Spermatogenesis is a prolonged process spanning 40–50 days in rodents. Manivannan et al. [61] showed that from 20 to 60 days the seminiferous tubules showed active developing stage with cellular units the sections exhibited rounded shape seminiferous tubules and many newly formed spermatogenic cells arranged properly inside the tubules At 60 days period all stages of spermatogenesis were clearly visible and spreaded in the lumen of the tubules as were seen in the corresponding control. The total duration of spermatogenesis, which takes approximately 4.5 cycles, lasts from 30 to 75 days in mammals [58, 62]. Although strain or breed differences can be found in the literature among members of the same species, the duration of the spermatogenic cycle has been generally considered constant for a given species. A recent study utilizing xenogenic spermatogonial transplantation has demonstrated that the spermatogenic cycle duration is under the control of the germ cell genotype [58].

In the present investigation, reduction in sperm number and motility was associated with an increase of sperm abnormalities in rats exposed to LN, which suggests the lead may impair the spermatogenesis or damage the genetic material of spermatogonia and spermatocytes by crossing the blood-testis barrier and gaining access to germinal cells. Furthermore, Hsu *et al*. [63] and Acharya *et al*. [57] declared that ROS generation causes chromosomal aberrations in germ cells by mutating certain gene segments involved in the maintenance of normal sperm structure, resulting in a deformed sperm population and/or drastically minimizing sperm count. The degraded sperm characteristics caused by lead administration may be due to a low testosterone concentration as observed in this study since a high level of testosterone is critically required for normal spermatogenesis, development, maintenance of sperm morphology and normal morphology and physiology of somniferous tubules [64]. In infertility and sterility, stem cell therapy promises to be a potential source of male and female germ cells. Not only embryonic stem cells (ESCs) but also fetal porcine skin stem cells, human fetal lung-MSCs, bone marrow and umbilical cord MSCs were the candidates for germ cell differentiation *in vitro*[53, 65]. Recently, stem cells experimentally derived from bone marrow have been used in experimental busulfan-treated infertility rodent models [66]. In the present study, the MSC group was associated with a significantly higher sperm count and motility, and a lower percentage of abnormal sperm population along with a concomitant increase in testosterone level, compared to the lead treated rat group. Hence, it could be concluded that MSCs might have a potential role in treating male infertility and testosterone deficiency. It could also be proposed that the beneficial effects of MSCs may be due to differentiation into male germ cells as reported by Nayernia *et al*. [67]. Yazawa *et al*. [68] proved that MSCs have the capacity to differentiate into steroidogenic cells, such as Leydig cells, both *in vivo* and *in vitro*. Lue *et al*. [69] showed that MSCs/BM, transplanted into testis of a busulfan-treated infertility mouse model, appeared to differentiate into germ cells, Sertoli cells and Leydig cells.

A growing amount of study has provided abundant evidence which has established the fact that metals are capable of interacting with nuclear proteins and DNA causing oxidative deterioration of biological macromolecules [9]. A decrease of total protein content was recorded in this study at different time intervals of lead treatment. Previously, Kansal *et al*. [70] reported a decrease in total protein level following LN exposure in mouse liver and kidney. They concluded that protein loss in lead toxicity might decrease the level of specific proteins such as albumin, hormones, hormone and metal binding proteins, enzymes and so on and thereby disturb the homeostasis and rate of metabolic activities.

Sperm DNA damage is a novel indicator of male infertility, which may be caused by an abnormal packaging and segregation of chromatin material, oxidative stress or abnormal cell apoptosis [71]. In the present study, genomic DNA alterations were estimated in epididymal sperm using RAPD profiles that reflect DNA effects in treated rats. Genetic changes in the rat genome included the obvious disappearance of the normal bands and appearance of new PCR products, indicated by the absence and presence of amplified RAPD fragments in DNA from lead exposed rats in comparison to those in the control DNA. The disappearance (deletion) or appearance (insertion) of an amplified RAPD fragment might be associated with DNA damage and mutations (for example, point mutations and large rearrangements) at the primer-template interaction sites, and/or unequal mitotic recombination or other effects (structural effects) which have facilitated primer hybridization [72]. In fact, participation of lead compounds in direct damage to DNA is not yet reported. However, evidence indicates that lead ions can apparently take part in a Fenton reaction to generate damaging oxygen radicals and can cause DNA strand breaks [73]. Also, some indirect mechanisms cause inhibition of DNA polymerase B, by lead induced ROS, possibly indicating the failure of DNA repair mechanisms [65]. In a study performed by Ahmed *et al*. [53], it was reported that lead treatment increased the percentage of chromosomal abnormalities in rabbit bone marrow cells.

During spermatogenesis, apoptosis in testicular germ cells is recognized as an important physiological mechanism to limit the germ cell population to numbers that the Sertoli cells can support [74]. In addition to its role in normal testicular physiology, apoptosis of germ cells has been recently reported as a mechanism responsible for the toxic damage to spermatogenesis [53]. Apoptotic DNA fragmentation observed in our study by gel electrophoresis after LN treatment was also reported in another study [75]. Such evidence may be due to a direct effect of Pb (II) on the DNA structure, oxidative mechanisms [76] or indirectly due to another mechanism involving the activation of caspases in the process of cell death [77]. In addition, impaired spermatogenesis is clearly depicted in the testicular histology. Exposure of adult male rats to lead can seriously alter the testicular tissues which started the changes with vacuolar degeneration until necrosis and atrophy of seminiferous tubules; the changes were time dependent according to the experimental groups. In addition, epididymal change in our results showed that some of the epididymal tubules were free of sperm which is considered an important contributory factor in infertility caused by lead. These findings support the results from other reports that lead alters the testes and reproductive tract in an animal model treated with lead [78].

It can be seen from our results that MSCs can ameliorate alteration in genomic DNA and protect the testis tissues from apoptotic damage induced by lead. Also, MSCs relatively improve the histopathological changes induced in the testis of rats. This protection is multifactorial, including modulating the oxidative stress reaction, tissue damage and repair. In this respect, El-Attar *et al*. [79] have reported that pretreatment with MSCs attenuates lipopolysaccharide-induced acute lung injury in rats through inhibition of neutrophilic recruitment, inflammation, oxidative stress and apoptosis. One theory of tissue repair holds that organ injury is ‘sensed’ by stem cells that migrate to the site of damage and differentiate into organ-specific cells, promoting structural and functional repair [80, 81]. LN administration induces severe damage to testicular cells, resulting in DNA and protein damage and apoptosis. Because these dead cells are not able to divide, other cells must replace them to repair the tissue and maintain organ homeostasis [82]. Cakici *et al*. [68] reported that MSCs were found both outside of the basal compartment and in the seminiferous tubules, supporting the idea that MSCs might have functioned in reestablishment of spermatogenesis in two ways: MSCs’ differentiation into sperm or maintenance of the spermatogonial stem cells. These results show that the MSCs could be both a rich and functional source for infertility treatment.

## Conclusions

In conclusion, the fertile status of LN treated male rats was recovered by treatment with MSCs in this study. Animals treated with MSCs showed mild degeneration in a few seminiferous tubules after 30 days. There was no histopathological alteration as recorded post 60 days of MSCs treatment, supporting the idea that MSCs might have functioned in the reestablishment of spermatogenesis by differentiation into sperm. MSCs have tremendous potential for regenerative medicine; MSCs/BM are capable of differentiating into germ cells and Leydig cells in the testis. In the present study, MSCs modulated the decline of serum testosterone levels induced by LN and approached within control values, especially at 60 days. Because Leydig cells are responsible for testosterone production, stem cell transplantation may replace the need of life-long testosterone supplementation in male hypogonadism. In addition, MSCs modulated DNA apoptosis in sperm and testicular tissues. These results show that MSCs could be both a rich and functional source for the treatment of infertility.

## Abbreviations

BM: bone marrow
BMP-2: bone morphogenetic protein-2
bp: base pair
CAT: catalase
(D)MEM: (Dulbecco’s) modified Eagle’s medium
dNTPs: deoxynucleotide triphosphates
EDTA: ethylenediaminetetraacetic acid
GPx: glutathione peroxidase
GR: glutathione reductase
LN: lead nitrate
MDA: malondialdehyde
MSCs: mesenchymal stem cells
RAPD: random amplified polymorphic DNA
RNase: ribonuclease
ROS: reactive oxygen species
RT-PCR: real time polymerase chain reaction
SDS: sodium dodecyl sulfate
SOD: superoxide dismutase
Taq DNA: Thermus aquaticus deoxyribonucleic acid
TGFβ3: transforming growth factor β3.
