# Erratum to: Evaluation of mesenchymal stem cells in treatment of infertility in male rats

**DOI:** 10.1186/s13287-017-0490-9

**Published:** 2017-02-09

**Authors:** Amal I. Hassan, Sally S. Alam

**Affiliations:** 1 Radioisotopes Department, Atomic Energy Authority, Giza, 12311 Egypt; 2 Cell Biology Department, National Research Center, El Tahrir Street, 12622 Dokki Giza, Egypt

## Erratum

The original article [1] omits a mention of a contribution to the research in the Acknowledgements section.

The authors would therefore like to acknowledge the role of Dr Laila Rashed in assisting with taking the pictures that constitute Figures 1B and 1D.

## References

[1] Hassan AI, Alam SS (2014). Evaluation of mesenchymal stem cells in treatment of infertility in male rats. Stem Cell Res Ther.

